# Population Status of a Cryptic Top Predator: An Island-Wide Assessment of Tigers in Sumatran Rainforests

**DOI:** 10.1371/journal.pone.0025931

**Published:** 2011-11-02

**Authors:** Hariyo T. Wibisono, Matthew Linkie, Gurutzeta Guillera-Arroita, Joseph A. Smith, Sunarto, Wulan Pusparini, Asriadi, Pandu Baroto, Nick Brickle, Yoan Dinata, Elva Gemita, Donny Gunaryadi, Iding A. Haidir, Herwansyah, Indri Karina, Dedy Kiswayadi, Decki Kristiantono, Harry Kurniawan, José J. Lahoz-Monfort, Nigel Leader-Williams, Tom Maddox, Deborah J. Martyr, Maryati, Agung Nugroho, Karmila Parakkasi, Dolly Priatna, Eka Ramadiyanta, Widodo S. Ramono, Goddilla V. Reddy, Ente J. J. Rood, Doddy Y. Saputra, Ahmad Sarimudi, Adnun Salampessy, Eka Septayuda, Tonny Suhartono, Ade Sumantri, Susilo, Iswandri Tanjung, Tarmizi, Koko Yulianto, Mohammad Yunus, Zulfahmi

**Affiliations:** 1 Wildlife Conservation Society - Indonesia Program, Bogor, Indonesia; 2 Forum HarimauKita, Bogor, Indonesia; 3 Fauna & Flora International, Cambridge, United Kingdom; 4 Durrell Institute of Conservation and Ecology, University of Kent, Kent, United Kingdom; 5 National Centre for Statistical Ecology, School of Mathematics, Statistics and Actuarial Science, University of Kent, Kent, United Kingdom; 6 Zoological Society of London - Indonesia Programme, Bogor, Indonesia; 7 Panthera, New York, New York, United States of America; 8 World Wide Fund for Nature-Indonesia, Jakarta, Indonesia; 9 Department of Fish and Wildlife Conservation, Virginia Tech, Blacksburg, Virginia, United States of America; 10 Fauna & Flora International - Indonesia Programme, Jakarta, Indonesia; 11 Indonesian Ministry of Forestry, Jakarta, Indonesia; 12 Leuser International Foundation, Medan, Indonesia; 13 Department of Geography, University of Cambridge, Cambridge, United Kingdom; 14 Rhino Foundation of Indonesia, Bogor, Indonesia; 15 Sumatran Tiger Conservation Program, Lampung, Indonesia; Smithsonian's National Zoological Park, United States of America

## Abstract

Large carnivores living in tropical rainforests are under immense pressure from the rapid conversion of their habitat. In response, millions of dollars are spent on conserving these species. However, the cost-effectiveness of such investments is poorly understood and this is largely because the requisite population estimates are difficult to achieve at appropriate spatial scales for these secretive species. Here, we apply a robust detection/non-detection sampling technique to produce the first reliable population metric (occupancy) for a critically endangered large carnivore; the Sumatran tiger (*Panthera tigris sumatrae*). From 2007–2009, seven landscapes were surveyed through 13,511 km of transects in 394 grid cells (17×17 km). Tiger sign was detected in 206 cells, producing a naive estimate of 0.52. However, after controlling for an unequal detection probability (where *p* = 0.13±0.017; ±S.E.), the estimated tiger occupancy was 0.72±0.048. Whilst the Sumatra-wide survey results gives cause for optimism, a significant negative correlation between occupancy and recent deforestation was found. For example, the Northern Riau landscape had an average deforestation rate of 9.8%/yr and by far the lowest occupancy (0.33±0.055). Our results highlight the key tiger areas in need of protection and have led to one area (Leuser-Ulu Masen) being upgraded as a ‘global priority’ for wild tiger conservation. However, Sumatra has one of the highest global deforestation rates and the two largest tiger landscapes identified in this study will become highly fragmented if their respective proposed roads networks are approved. Thus, it is vital that the Indonesian government tackles these threats, e.g. through improved land-use planning, if it is to succeed in meeting its ambitious National Tiger Recovery Plan targets of doubling the number of Sumatran tigers by 2022.

## Introduction

Setting conservation priorities for top predators requires repeatable and robust estimates of abundance or distribution over large areas. These assessments should be conducted at a meaningful scale for the species in question, such as a landscape, sub-species distribution, or overall species range [1]. For conservation managers working in tropical rainforests, obtaining such estimates for these taxa are difficult because they tend to be cryptic and live at low densities across large areas [2], [3]. This situation is pertinent to Sumatran rainforests that support several iconic and highly threatened wildlife species, such as the tiger.

The main strategy followed to conserve Sumatran wildlife and its habitats has been to establish large protected areas, including the mountainous national parks of Kerinci Seblat (13,971 km^2^) and Gunung Leuser (7,927 km^2^). However, extensive tracts of lower elevation forests were excised during their designation to allow for commercial logging. These lowland forests can support relatively high densities of Sumatran tigers and act as important corridors that maintain landscape integrity and therefore population viability [4]. Unfortunately, conservation investment has tended to overlook unprotected lowland forests across Sumatra and, as a result, these highly accessible rainforest habitats (<150 m) have come under immense pressure and are experiencing disproportionately high rates of deforestation (3.3%/yr; [5]).

Reliable information is required on the conservation status of flagship species to better understand the impact of deforestation on Sumatra's wildlife. For the Sumatran tiger, previous population assessments have fixated on estimating the total number of individuals across the island or within several protected areas [6], [7]. However, these estimates have failed to adequately control for unequal detection probabilities or they have extrapolated abundance estimates recorded from a few areas to the entire island, thereby limiting their reliability and, in turn, usefulness for setting management priorities (e.g. [8]). In this study, we aim to conduct the first rigorous assessment of the Sumatran tiger population status across its entire island range. We use methods that explicitly account for imperfect and heterogeneous detection to model species occupancy and investigate the influence of biophysical and anthropogenic threat covariates on this state variable.

## Methods

### Ethics statement

We would like to thank the Indonesian Ministry of Forestry for their permission to conduct this work and for the support of the Director of Biodiversity Conservation in its implementation.

### Field surveys

From 2007–2009, eight organisations (Wildlife Conservation Society, Fauna & Flora International, Durrell Institute of Conservation and Ecology, World Wildlife Fund, Zoological Society of London, Sumatran Tiger Conservation and Protection, Leuser International Foundation, Rhino Foundation of Indonesia and the Sumatran Tiger Protection and Conservation Foundation) partnered with the Indonesian Ministry of Forestry to conduct simultaneous field surveys across Sumatran rainforest under different management regimes. The survey protocol was developed from a detection/non-detection sampling framework proposed by MacKenzie *et al.* [9] to study occupancy that has been previously applied to tigers [4], [10].

Within each cell, a team of 4–5 people surveyed locations considered likely to contain tiger pugmarks, e.g. ridge trails. The locations of tiger detections were recorded with a GPS along transects within 17×17 km grid cells. Cell size was based on the putative home range size of an adult male Sumatran tiger to allow changes in the distribution of resident tigers to be reflected as changes in the proportion of the grid cells occupied. Survey teams aimed to achieve wide spatial coverage of each cell, but this was influenced by the prevailing topography. For example, such coverage was less easy to achieve in rugged mountainous cells where deviations from pronounced ridge trails required descending steep slopes, often for hundreds of metres. Surveys were conducted in all habitat types likely to support tigers, from sea-level peat swamp to forests around the volcanic peak of Mount Kerinci, the highest point on Sumatra (3,805 m asl). In total, 13,511 km of transects were surveyed in 394 cells that covered seven landscapes across all eight mainland Sumatran provinces (Table 1).

**Table 1:** Summary of Sumatra-wide field survey effort for each landscape.

Study area	TCLstatus*	SurveyDates	Average yearly forest loss (%)	# grid cells	
				Surveyed	with tiger sign
Kerinci Seblat-Batang Hari^1^	I	09/01/07–10/09/09	0.8	110	76
Southern Sumatra^2^	II+III	24/03/07–25/06/08	1.2	51	21
Way Kambas National Park	-	06/01/08–11/03/08	2.3	10	2
Leuser-Ulu Masen	I	02/05/07–01/03/09	0.8	159	76
Northern Riau^3^	n/a	09/06/09–22/12/09	9.8	18	0
Central Sumatra^4^	I+II+III	09/04/07–15/10/09	1.9	31	21
Eastern Sumatra^5^	n/a	26/04/07–21/11/09	2.2	15	10

*I = global priority; II = regional priority; III = long-term priority.

1 Kerinci Seblat National Park and Batang Hari Protection Forest and their surrounding forests.

2 Bukit Barisan Selatan National Park and Bukit Balai Rejang Selatan.

3 Pasir Pangaraian, Giam Siak, Duri, Balaraja, Tapung.

4 Tesso Nilo, Bukit Bungkuk, Bukit Rimbang-Baling, Bukit Batabuh, Bukit Tigapuluh, Kerumutan.

5 Dangku, Bukit Duabelas, Berbak.

### Database compilation

To match the discrete sampling protocol assumed by the models used, in which a number of replicate surveys are conducted within each sampling site, transects were divided into segments, assigning ‘1’ to those containing at least one detection and ‘0’ otherwise. In order to account for variation in terrain ruggedness, distances were determined by overlaying the two-dimensional tracklogs from GPS handsets carried by field teams onto a three-dimensional digital elevation model.

Tiger site occupancy was considered to vary across Sumatra, given the island's diverse topographic composition, ranging from prey-rich lowland forests to less productive and rugged montane forests. Furthermore, the influence of anthropogenic threats on habitat quality was expected to negatively affect tiger occupancy. Deforestation was considered to be important because Sumatra has one of Southeast Asia's highest rates of conversion from intact forest to non-forest (WWF-Indonesia 2010). To explore the influence of biophysical and anthropogenic threat covariates on tiger occupancy, a spatial dataset of nine potential explanatory variables was constructed within ArcGIS v9.3 software (ESRI). Information was obtained from several sources: elevation and slope [11]; distance to roads and to settlements (Indonesian National Coordination Agency for Surveys and Mapping); grid cell protection status (mostly inside or outside a protected area; Ministry of Forestry); and, distance to a forest patch, distance to forest edge (from within the forest), percentage of forest cover and deforestation [5]. Elevation, slope and distance covariates were extracted at a 30×30 m resolution and a single value per site was obtained by averaging all the pixel values within each site. Deforestation was defined as the area (ha) of forest cover that had been completely removed between 2000 and 2008.

### Data analysis

Tiger detection/non-detection data were analyzed to estimate site occupancy (ψ) using models that explicitly account for imperfect species detection: the basic occupancy model [9] and three of its extensions. The first extension (‘clustering model’) relaxes the assumption of independence among replicates and models first-order Markovian dependence between consecutive replicates [12]. This was considered relevant because data were collected along transects and detections in consecutive transect segments might not always be independent. The second extension (‘beta-binomial model’) relaxes the assumption of no unexplained variation in detection probability across sampling sites and models heterogeneity using a beta distribution for the continuous mixture on detection probabilities [13]. The third extension (‘abundance model’), provides an alternative mixing distribution to accommodate heterogeneous detection probabilities based on a structure constructed to model abundance-induced heterogeneity [13]. For the discrete mixture that describes species site abundance, a Poisson distribution with parameter λ and two of its generalizations to allow for zero-inflation and overdispersion (i.e. negative-binomial) were used. Subject to the assumptions of the model being met (see review of this in *Discussion*), the mean of the estimated mixing distribution (e.g. λ for a Poisson) may be interpreted as an estimate of average site abundance. Site occupancy is not a formal parameter in the formulation of the abundance model and was derived as the probability of having at least one individual given the estimated abundance distribution, e.g. ψ = P(*N_i_*>0) = 1-exp(−λ), if a Poisson).

Candidate explanatory variables for tiger site occupancy/density were standardized using a z-transformation and assessed for collinearity. Two pairs of variables showed strong significant correlation (Pearson's r = 0.80 for elevation and slope; r = −0.78 for forest cover and distance to forest) and were not included together within the same models. Tiger detection history was constructed by defining the survey replicates as 5 km transect segments. This was chosen to mitigate the dependence between consecutive replicates that, given tiger movement patterns, could be expected at smaller scales, but without compromising the results by the loss of data that would result when choosing a very coarse replicate length. To assess the robustness of the results to moderate changes in the definition of replicates, models were also run using different segment lengths (4 and 6 km).

The analysis was performed obtaining maximum-likelihood estimates by numerical maximization, using RMark 2.0.1 for the basic occupancy and abundance (Poisson and negative binomial) models and Matlab scripts for the clustering, beta-binomial and abundance (zero-inflated Poisson) models. The model selection procedure used AICc to compare model fit, with the effective sample size defined as the number of sampling sites. For the best model, individual site estimates of the real parameters were derived from the regression coefficient estimates (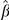), as well as conditional estimates given the observed data, which were then averaged for each study area.

## Results

### Model selection procedure

Tiger signs were detected in 206 of 394 cells, corresponding to a naïve occupancy estimate of 0.52. The model that best explained the observed data was a Poisson abundance model dependent on average distance to forest, elevation, recent deforestation and protected area status. This model had much stronger support than the constant model (ΔAICc = 61) and was considerably better than the best competing model with one covariate less (ΔAICc = 7.5). Adding one extra covariate only marginally improved model fit and the confidence interval of the corresponding regression coefficient included zero. There was no support for zero-inflation in the abundance distribution while models that allowed for overdispersion did not provide better fit or failed to converge. The basic, clustering and beta-binomial models were poor fits to the data in comparison to the abundance model. Moderate variations in the segment length used to define the spatial replicates did not lead to substantial changes in the results. The same model provided the best explanation for the data and the support of the next highest ranked models remained consistent.

### Status and threats of Sumatran tigers

**Figure 1:**
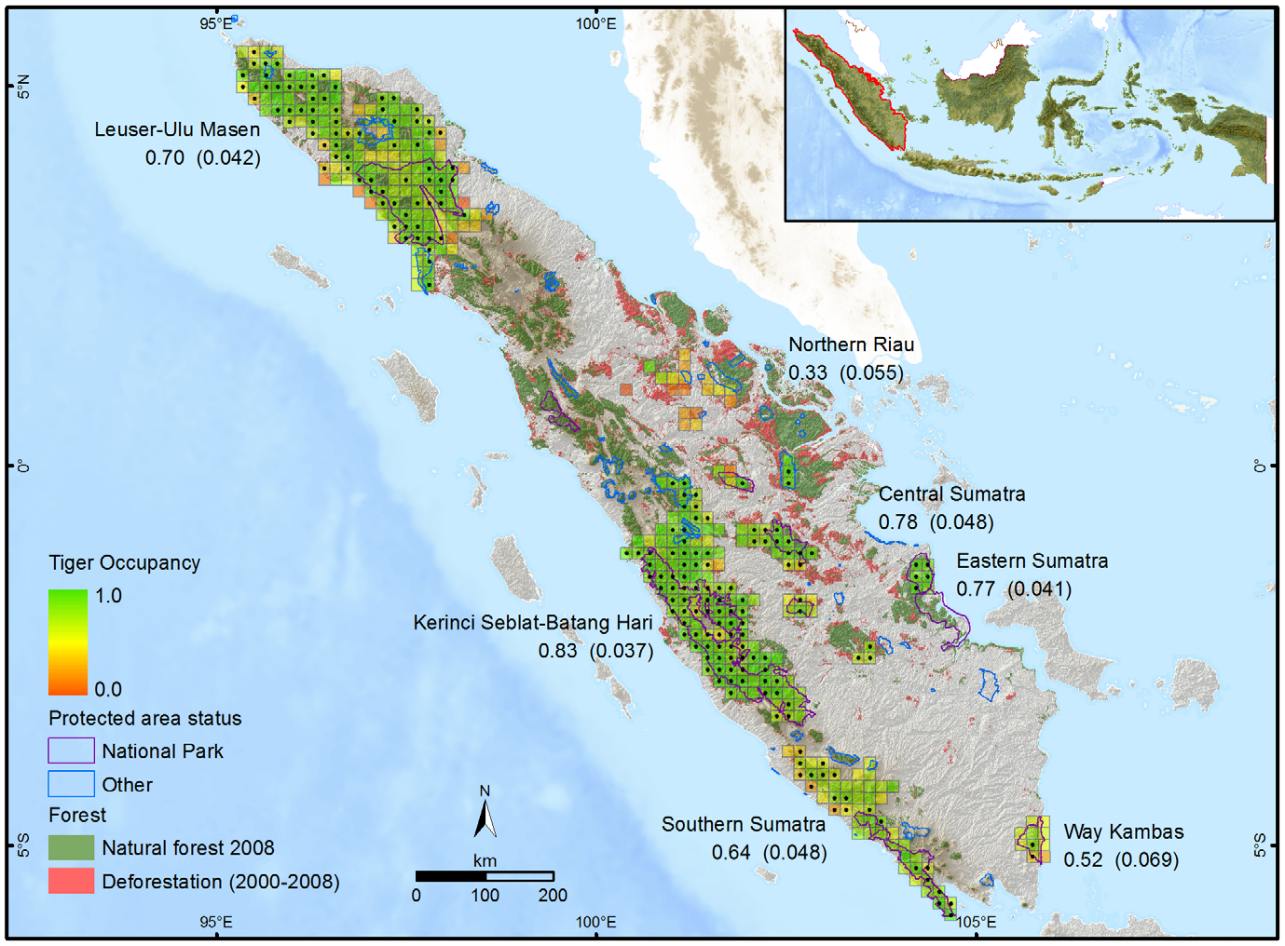
Estimated Sumatran tiger site occupancy 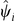 and sites with actual indirect tiger sign detections (black dots).

Tiger occupancy estimates varied considerably within the different landscapes (Figure 1; Table 2), and was highest for the large Kerinci Seblat-Batang Hari landscape (0.83±0.037; 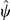
±SE). While estimated occupancy was relatively low for some of the smaller landscapes, e.g. Way Kambas National Park (0.52±0.069), it was high for others, e.g. Eastern Sumatra (0.77±0.041). The lowest estimate corresponded to the Northern Riau landscape (0.33±0.055), which has undergone the highest rate of recent forest conversion on Sumatra. For the 3.3 million ha contiguous Leuser-Ulu Masen landscape, the occupancy estimate (0.70±0.042) provides the first reliable tiger conservation status evaluation for Sumatra's largest continuous forest estate, which is now recognised as a global priority for wild tiger conservation (GTI 2010).

**Table 2:** Sumatran tiger site occupancy (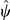) and density (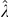) estimates for the best model, averaged for each study area.

Study area	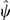	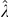	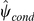	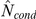
Kerinci Seblat-Batang Hari	0.83 (0.037)	2.0 (0.28)	0.88 (0.021)	2.2 (0.27)
Southern Sumatra	0.64 (0.048)	1.2 (0.16)	0.63 (0.041)	1.0 (0.13)
Way Kambas National Park	0.52 (0.069)	0.8 (0.15)	0.45 (0.055)	0.6 (0.10)
Leuser Ecosystem-Ulu Masen	0.70 (0.042)	1.4 (0.19)	0.69 (0.035)	1.3 (0.17)
Northern Riau	0.33 (0.055)	0.5 (0.09)	0.16 (0.038)	0.2 (0.05)
Central Sumatra	0.78 (0.048)	1.7 (0.27)	0.80 (0.027)	1.8 (0.23)
Eastern Sumatra	0.77 (0.041)	1.9 (0.30)	0.67 (0.025)	1.8 (0.25)
Overall	0.72 (0.039)	1.5 (0.20)	0.71 (0.030)	1.5 (0.19)

The two right-most columns show the estimates conditional to the data observed. Standard errors are shown in brackets.

The occupancy estimate of Sumatran tigers across the entire island was 0.72(±0.039), with an average estimated 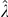 of 1.5(±0.20) and individual detection probability 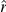 of 0.13(±0.017). The regression coefficients on λ in the best model suggest that tiger density, and therefore occupancy, was higher in habitat that was at lower elevation 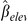 = −0.23 (±0.073), closer to forest patches 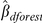 = −0.63 (±0.116), with less recent forest clearance 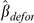 = −0.28 (±0.085), and within protected areas 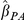 = 0.39 (±0.125). The corresponding estimated odd ratios for a one unit increase in each of the covariates 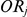 = 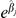
are 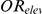 = 0.79 (±0.058), 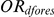 = 0.532 (±0.062), 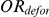 = 0.756 (±0.064), and 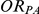 = 1.477 (±0.185).

## Discussion

Managers require population estimates that cover meaningful units, whether at landscape, sub-species or species scales. However, gaining such information for cryptic species living at low densities across large areas has previously proved difficult. This study provides the first comprehensive assessment of Sumatran tigers using site-specific surveys and modelling detection probability. We believe there is cause for optimism for the long-term survival of Sumatran tigers because from the major landscapes surveyed, the species still has a reasonably good conservation status. However, surveys tended to be conducted in prime habitat and with poorer habitat, with presumably lower occupancy, generally being excluded. Our results reveal the insidious effects of deforestation, especially in the patchier forests that had occupancy levels which were 20% lower than the island average. Thus, maintaining forest integrity is critical for the long-term survival of tigers, especially given the road expansions planned through the core tiger areas of Kerinci Seblat National and the Leuser Ecosystem. For landscapes that are already fragmented, such as those in Riau, reconnecting forest blocks is recommended. However, given the rapid conversion of remaining forests in this province, stopping further fragmentation and maximizing chances for tiger dispersal between remaining forest blocks would be considered a significant achievement in itself.

The sampling protocol implemented in this study has wide application to other difficult to detect species. However, there are several caveats associated with the use of the abundance model that ranked top in our analysis. Understanding how underlying model assumptions are met is essential to the correct interpretation of estimates obtained. The model assumes that differences in abundance are the only source of heterogeneity in site-specific detection probabilities; otherwise bias may be induced in the estimators. In this study, a well-defined protocol was developed and implemented to minimize heterogeneity in detection probability. Nevertheless, some residual, unmodelled heterogeneity may still have remained, e.g. pugmark detections tended to be easier in wetter substrates and surveys were conducted over both wet and dry seasons. The model also assumes that tiger site abundance closely fits a Poisson distribution. Tigers are territorial so it could be expected that their distribution exhibits some degree of under-dispersion. Finally, the model is based on a functional dependence between species detectability 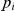 and the number of individuals 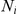 at a site of the form 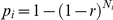 where *r* is the individual detectability. This relationship implies that all individuals within each site are equally detectable at any replicate survey (i.e. transect segment), i.e. the system is closed to changes in abundance. The impact of departures from the closure assumption in occupancy studies with spatial replication has received attention recently for the basic occupancy model [14], but has not been explored for abundance models. In our survey, sites were larger than female tiger territories, which tend to exhibit little overlap [15]. The assumption of equal detectability in any replicate would therefore be violated. However, a relationship of increased detection probability with increased abundance can still be expected, with greater tiger numbers resulting in an increase in the area within a site covered by their territories, where the species is detectable. When dealing with few individuals per site and low detectability, the functional relationship assumed by the model is well approximated by a linear function 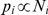, which would be compatible with a scenario of site coverage proportional to abundance. Therefore, although we are cautious about interpreting our estimates as absolute numbers, we believe they provide a valuable tool to assess differences in relative abundance across the landscape and their relationship to environmental and anthropogenic factors.

This study overcame three limitations associated with previous assessments of Sumatran tiger; unmodelled detection probability, uncontrolled confounding variables and lack of site-specific survey data. Thus, the state variable (occupancy) was estimated from tiger sign data while accounting for detection probabilities and the influence of several biophysical and anthropogenic threat covariates. Next, the rapid survey technique, which only required spatial replicates within a sampling unit rather than temporal replicates that would have required multiple visits to the cell, enabled the majority (58%) of presumed tiger habitat to be covered [3]. A final and peripheral limitation, i.e. poor coordination between NGOs, was also overcome during this study.

To complete the Sumatra-wide survey, 13,511 km of transects were walked. To put this effort into context, Sumatra is 1,790 km in length. Whilst our island-wide survey provides the first baseline data and first monitoring system that have been urgently requested by the Global Tiger Initiative for Indonesia [16], it is recommended that a future island-wide survey be conducted at five year intervals, i.e. to evaluate the National Tiger Recovery Plan goals [17]. Prior to this, a revision of the sampling design, with a view to greater cost-effectiveness, should be made, whereby the number of sampling cells and occasions needed to achieve a desired level of precision are predetermined.

The surveys conducted in this study primarily focussed on protected areas that are recognised as being priority (or the most important) landscapes where tiger occupancy should therefore be highest. Expanding the surveys to cover the 42% remaining tiger landscapes, where deforestation and fragmentation is higher [3], may lower the overall occupancy value. These forests are important because they act as tiger corridors, e.g. between Kerinci Seblat and Batang Hari, and therefore enable gene flow between subpopulations and increased genetic viability. Their protection will require inter-provincial government and inter-agency collaborations, e.g. the Ministry of Interior who coordinates between provincial and local government and between the Ministries of Forestry (which manages forests) and Public Works (which manages infrastructure development). Thus, the high profile declaration made at the 2008 IUCN World Conservation Congress by all ten Sumatran provincial governors, with endorsements from four Ministers, to coordinate regional-wide spatial planning and ecosystem restoration was an unprecedented and positive step [18]. However, it has not translated into on-the-ground action. For example, the Government of Riau continued its fast-tracked economic development plan that prioritises the conversion of forest estates with oil palm and pulp/paper wood plantations and which has over 25 years resulted in 65% of the province's forest being cleared [5]. District governments within Bengkulu and Jambi provinces have submitted road construction proposals, and allocated annuals budgets, that would bisect Kerinci Seblat National Park in three core areas [19].

Whilst the status of Sumatran tigers was good in the major landscapes, the province of Riau, where there were fewer tigers detections, provides a sobering reminder of what can happen if high deforestation rates, and the associated conflicts between people and tigers, are not mitigated [20]. In stark contrast and as an example to other provinces, the Government of Aceh has implemented a logging moratorium that has, since 2007, ceased all commercial logging, as well as the development of oil palm and other agricultural plantations [21]. Here, the government has prioritised an economic development strategy that aims to generate revenue from managing intact forest through Reducing Emissions from Deforestation and Forest Degradation (REDD) initiatives. Complementing a traditional protected area management strategy with such an innovative approach to forest management, as planned under the Indonesia-Norway Letter of Intent [22], will be needed if the Ministry of Forestry is to meet its long-term strategic goal of doubling the Sumatran tiger population by 2022 [17].
